# Phytol-mixed micelles alleviate dexamethasone-induced osteoporosis in zebrafish: Activation of the MMP3–OPN–MAPK pathway-mediating bone remodeling

**DOI:** 10.1515/biol-2022-1015

**Published:** 2025-03-21

**Authors:** Bo Liu, Peng Wang, Xiangyang Lv

**Affiliations:** Department of Trauma Surgery, Hebei Port Group Co LTD. Qinhuangdao Hospital of Integrated Chinese and Western Medicine, Qinhuangdao, Hebei, 066003, China; Department of Spine Surgery, Shengli Oilfield Central Hospital, Dongying, Shandong, 257000, China; Department of Orthopedics, Xi’an Qinhuang Hospital, Xi’an, Shaanxi, 710600, China

**Keywords:** phytol, osteoporosis, MMP3-OPN-MAPK pathway, zebrafish

## Abstract

This research investigates the therapeutic efficacy of phytol-mixed micelles in mitigating dexamethasone (Dex)-induced osteoporosis in zebrafish, with a particular focus on scale regeneration. Osteoporosis was induced in zebrafish through exposure to Dex, and the effects of phytol-mixed micelles were evaluated in this model. Following phytol therapy, bone mineralization was assessed using calcium, phosphorus, and alizarin red staining tests. Additionally, commercially available kits quantified the levels of tartrate-resistant acid phosphatase (TRAP), hydroxyproline (HP), and alkaline phosphatase (ALP). The mRNA expression levels of MMP3, osteopontin (OPN), and mitogen-activated protein kinase (MAPK) were examined using reverse transcription polymerase chain reaction (RT-PCR). The findings indicated that phytol significantly increased calcium and phosphorus concentrations. Phytol-mixed micelle therapy led to increased calcium deposition and enhanced bone formation, as evidenced by alizarin red staining. Moreover, phytol administration resulted in increased HP content and upregulated ALP and TRAP activities in zebrafish. RT-PCR tests demonstrated that phytol plays a role in the restoration of the MMP3–OPN–MAPK pathway. In summary, this research highlights the potential of phytol-mixed micelles in ameliorating Dex-induced osteoporosis in zebrafish. Clarifying phytol’s mechanism, particularly its stimulation of the MMP3–OPN–MAPK pathway, provides insight into its role in facilitating bone remodeling.

## Graphical abstract

**Figure:**
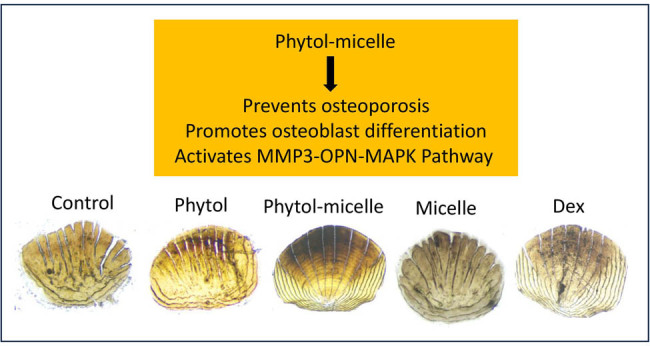

## 1 Introduction

Osteoporosis, a pervasive chronic bone condition characterized by decreased bone density and structural integrity, is a serious public health concern that affects the quality of life for millions of people worldwide [1]. The onset of osteoporosis is influenced by a wide range of intrinsic and extrinsic factors, including aging, hormonal fluctuations, a sedentary lifestyle, inadequate intake of essential nutrients such as calcium and vitamin D, exposure to certain medications like glucocorticosteroids, and genetic predispositions [2].

The preservation of bone mass and integrity depends on the delicate balance between bone creation and bone resorption, regulated by osteoclasts, osteoblasts, and osteocytes [3]. In the process of bone remodeling, osteoclasts, responsible for bone resorption, and osteoblasts, engaged in bone production, play vital roles, ensuring that skeletal tissue is continuously renewed and corrected. The development and progression of osteoporosis are marked by disruptions in this delicate equilibrium, occurring when bone resorption exceeds bone production [3]. Conversely, reduced osteoclastic activity can result in bone mass accumulation, leading to disorders such as osteosclerosis. To effectively manage bone disorders, it is essential to understand the complex processes regulating the physiological and pathological functions of osteoclasts.

One of the most important mediators of osteoclast development and activation is the receptor activator of nuclear factor-κB ligand (RANKL), a member of the tumor necrosis factor family of ligands. The binding of RANKL to its receptor RANK on osteoclasts activates signaling cascades that enhance osteoclast maturation and activity, facilitating bone resorption [1,2,3,4]. Osteoprotegerin (OPG), a decoy receptor for RANKL, acts as an antagonist to this process by preventing the interaction between RANKL and RANK, thereby limiting osteoclastogenesis and bone resorption. The RANKL/RANK/OPG pathway is thus crucial in the control of bone remodeling and presents potential therapeutic intervention targets [5].

Recent research has highlighted the therapeutic potential of targeting the RANKL pathway in the treatment of osteoclast-mediated bone loss diseases. Clinical trials have shown that RANKL inhibitors, such as denosumab, are effective in reducing bone remodeling, increasing bone mineral density, and lowering the incidence of fractures in postmenopausal women with osteoporosis [6,7]. Additionally, substances like melatonin and OPG have shown potential in preventing bone loss by inhibiting the RANKL pathway [8,9]. Estrogen, known to modulate bone resorption by increasing OPG levels, also holds promise as a potential therapeutic agent [10].

Despite progress in osteoporosis treatment, there remains a need for safer and more effective therapeutic methods. Most current medications aim to prevent bone resorption by decreasing osteoclast activity; bisphosphonates are among the most commonly prescribed. However, long-term use of bisphosphonates is associated with several adverse effects, including gastrointestinal issues and jaw bone necrosis, necessitating the exploration of alternative treatments [11,12,13].

Investigating the therapeutic potential of natural compounds like phytol, which possesses various pharmacological properties such as antioxidant and anti-inflammatory actions, offers promise for developing novel osteoporosis treatments [12]. The potential of natural compounds to improve bone health and reduce bone loss has made them attractive choices for treating osteoporosis. Through their modulation of bone remodeling processes, compounds including genistein, resveratrol, and curcumin have proven anti-osteoporotic properties. For example, studies have shown that curcumin inhibits osteoclastogenesis and increases osteoblast activity, whilst resveratrol improves bone mineral density and lowers inflammation. It has been noted that the phytoestrogen genistein enhances bone strength and density. It is necessary to do more studies and clinical trials to confirm the safety and effectiveness of these natural chemicals, which provide a less harmful, more comprehensive approach to controlling osteoporosis [12,13,14]. However, there is a paucity of research on the effects of phytol on bone health, underscoring the need for further studies in this area. Elucidating the molecular mechanisms regulating osteoclasts, particularly the RANKL/RANK/OPG pathway, is essential for expanding our understanding of osteoporosis pathogenesis and identifying novel treatment targets. By leveraging insights from these pathways and exploring new therapeutic approaches, we aim to address the urgent need for more effective and safer osteoporosis treatments.

## 2 Materials and methods

### 2.1 Phytol mixing in micelles

To begin the production of phytol-mixed micelles, 1 g of phytol was dissolved in 10 mL of chloroform. Next, 0.2 mL of this solution was added to 3.8 mL of chloroform containing various concentrations of lipids (EPC and DSPE-PEG 2000; 50:50). The solvent was then evaporated under vacuum at a temperature of 60°C for 20 min. Concurrently, 110 mg of glycocholic acid hydrate (0.24 mmol) was dissolved in 8 mL of 0.067 M phosphate buffer at 60°C. This solution was applied to the lipid film made of EPC, DSPE-PEG 2000, and phytol to hydrate it. After magnetic stirring for a minimum of 4 h at room temperature, a clear dispersion was obtained. This dispersion was further processed by being extruded three times through a syringe filter.

To investigate the phytol-loading capacity of the DSPE-PEG/EPC 50/50-mixed micelles, DSPE-PEG (288 mg, 0.10 mmol) and EPC (80 mg, 0.10 mmol) were dissolved in 4 mL of chloroform in a round-bottom flask. Subsequently, either 0.4 or 0.8 mL of phytol (100 mg/mL in chloroform) was mixed, and the process was repeated as described until a clear dispersion was achieved. To prevent filter occlusion, the micellar dispersions were diluted tenfold with a 0.067 M phosphate buffer before filtration.

### 2.2 Cell culture

The MG63 immortalized human osteoblast cell line was cultured in Dulbecco’s modified Eagle’s medium supplemented with 10% fetal bovine serum under standard conditions (37°C and 5% CO_2_) to ensure optimal growth and viability. Cellular activity was assessed using the 3-(4,5-dimethylthiazol-2-yl)-2,5-diphenyltetrazolium bromide (MTT) assay, following a previously established protocol [15]. Cells were seeded at a density of 2 × 10^4^ cells per well in 96-well plates and allowed to adhere and proliferate for up to 12 h. Subsequently, the cells were exposed to varying concentrations of phytol-micelles (0–50 μg) for up to 48 h. After the specified exposure time, an MTT solution (5 mg/mL) was added to each well, and the plates were incubated at 37°C for an additional 4 h, allowing viable cells to convert the yellow MTT dye into purple formazan crystals via mitochondrial activity. The formazan crystals were then solubilized using an appropriate solvent, and the absorbance of the resulting solution was measured at 570 nm using a microplate reader. The absorbance readings provided a quantitative measure of cellular activity. Additionally, alkaline phosphatase (ALP) activity and alizarin red staining assays were conducted as outlined in a previous study [15], providing further insights into the osteogenic potential and mineralization capacity of the treated cells. These complementary assays offered comprehensive information regarding the effects of phytol-micelles on osteoblast cellular activity and bone formation processes.

### 2.3 Quantification of osteonectin (ON) and osteocalcin (OC)

To quantify ON and OC levels, an enzyme-linked immunosorbent assay (ELISA) was conducted using an ELISA kit from Elabscience following the manufacturer’s instructions. A conditioned medium was obtained following the treatment of cells with phytol-micelles. The ELISA procedure began by adding specific volumes of the conditioned medium, along with appropriate diluents or reagents, to the wells of a microplate pre-coated with antibodies specific to ON and OC. After an incubation period allowing ON and OC in the conditioned medium to bind to the immobilized antibodies, the microplate was washed to remove any unbound substances. Next, enzyme-conjugated secondary antibodies were added to the wells, specifically binding to the captured ON and OC. Following another washing step to eliminate excess secondary antibodies, a substrate solution was added, triggering a colorimetric reaction. The intensity of the resulting color was measured spectrophotometrically at a specific wavelength, and the absorbance values were recorded. These absorbance values were then used to determine the concentrations of ON and OC in the conditioned medium by comparing them to a standard curve generated using known concentrations of ON and OC standards provided in the kit. This process facilitated the accurate quantification of ON and OC levels in the conditioned medium, thereby enabling the assessment of the effects of phytol-micelles on the secretion of these osteogenic markers by the cells.

### 2.4 Animal maintenance and scale regeneration model

The experiment involved maintaining 6-month-old zebrafish at 28°C with a light cycle of 14 h and a dark cycle of 10 h in an aquarium with regular fresh water exchange. The fish were fed live brine shrimp and commercial flake food three times daily. To induce osteoporosis, 20 adult zebrafish were treated with dexamethasone (Dex) at a concentration of 10 μM. Following osteoporosis induction, the fish were subjected to various doses of phytol-micelles, ranging from 0 to 50 μM, to evaluate its impact on bone production and scale regeneration. Thirty scales were surgically removed from each fish on the first day after Dex treatment, taken from below the operculum to the center of the first dorsal fin. The fish were then maintained under the specified treatment conditions for 14 days. At the end of the treatment period, the regenerated scales were carefully collected for further analysis. The experimental protocol followed established in-house procedures and techniques previously published [15].


**Ethical approval:** The research related to animal use has been complied with all the relevant national regulations and institutional policies for the care and use of animals.

### 2.5 Calcium and phosphorus ratio analysis by inductively coupled plasma optical emission spectrophotometry (ICP-OES)

The mineral content, specifically calcium and phosphorus, in the scale samples was tested following a series of steps. Initially, and the scales were freeze-dried until completely dehydrated. The dried materials were then dissolved in 70% nitric acid (Sigma-Aldrich) to disintegrate the organic matrix and extract the mineral components. The dissolved samples were diluted 100-fold with demineralized water for analysis. The diluted samples were analyzed using ICP-OES, an analytical method that accurately measures calcium and phosphorus levels by analyzing their emission spectra. This provided precise and reliable data on the mineral composition of the scale samples.

### 2.6 Hydroxyproline (HP) assay

Scale samples were collected and thoroughly washed with saline to remove external contaminants and then dried at approximately 80°C for 48 h until a constant weight was achieved. The dried samples were incinerated in a silica crucible at 800°C to remove any remaining organic material, leaving behind only the mineral components. The weight of the pulverized samples was accurately recorded. The samples were then hydrolyzed using 6 mol/L hydrochloric acid (HCl) to break down collagen fibers into their constituent amino acids, including HP. The concentration of HP was determined using a standardized protocol provided by the assay kit manufacturer, likely involving colorimetric or spectrophotometric methods. The absorbance of the chromophore produced during the reaction between HP and specific reagents was measured, and the absorbance values were correlated with known HP concentrations to quantify its presence in the samples. This assay provided valuable information about the collagen content in the scale samples, essential for assessing bone health and integrity.

### 2.7 ALP activity

To determine ALP activity, the following procedure was followed. Fixed scales were carefully cut and pretreated with an alkaline buffer solution consisting of 100 mM Tris–HCl (pH 9.5), 1 mM MgCl_2_, and 0.1 mM ZnCl_2_. The scales were incubated in this alkaline buffer for 30 min for proper conditioning. After pretreatment, the scales were incubated with 150 μL of alkaline buffer containing 20 mM 4-nitrophenyl phosphate disodium salt hexahydrate (pNPP, Sigma) for 1 h. pNPP serves as a substrate for ALP, and its hydrolysis results in the formation of a yellow-colored product. Following the incubation period, the enzymatic reaction was halted by adding a solution containing 3 N NaOH and 20 mM ethylenediaminetetraacetic acid, effectively stopping further enzymatic activity and stabilizing the reaction mixture. The ALP activity was quantified using a spectrophotometric method at a wavelength of 405 nm. The absorbance of the reaction mixture was measured, with the intensity of the yellow color formed being directly proportional to the ALP activity present in the sample. This procedure accurately determined the ALP activity in the scales, providing valuable insights into the bone metabolism and health of the specimens.

### 2.8 Tartrate-resistant acid phosphatase (TRAP) activity

To assess TRAP activity, the protocol outlined in Persson et al. [16] was followed with slight modifications. Initially, fixed scales were immersed in a solution containing 0.1 M sodium acetate buffer supplemented with 20 mM tartrate and incubated for 1 h. Subsequently, the solution was replaced with a fresh one consisting of 20 mM tartrate, 0.1 M sodium acetate buffer, and 20 mM pNPP, allowing the enzymatic reaction to proceed and facilitating the hydrolysis of pNPP. After incubation, the reaction was halted by adding 50 μL of 2 N NaOH, ensuring no further hydrolysis occurred. The amount of para-nitrophenol (pNP) produced, indicative of TRAP activity, was quantified by measuring the absorbance at 405 nm using a spectrophotometer. These absorbance data were then used to calculate the concentration of pNP produced, employing a standard curve specifically generated for pNP.

### 2.9 Alizarin red staining

For Alizarin red staining, zebrafish scales were first collected and fixed overnight in a 4% paraformaldehyde solution. Following fixation, the scales were washed with 10% glycerol/0.5% KOH solution to remove excess fixative. Subsequently, the fish larvae were stained overnight with 0.02% Alizarin red stain/10% glycerol/0.5% KOH solution to visualize the formed bone. After staining, the scales were destained using 50% glycerol/0.5% KOH solution to remove any excess stain and enhance clarity. A bleaching step was then performed using 3% H_2_O_2_/0.5% KOH solution to remove background interference. Finally, digital photographs of the stained scales were acquired using a fluorescence stereomicroscope for further analysis and documentation.

### 2.10 Real-time RT-PCR analysis

Total RNA was extracted from the scales using TRIzol reagent (Invitrogen, USA) following the manufacturer’s protocol. Immediately after extraction, RNA samples were assessed for purity and concentration using a NanoDrop 2000 spectrophotometer (Thermo Fisher Scientific, USA). To prevent RNA degradation, samples were aliquoted and stored at −80°C until further analysis. For sample preparation, care was taken to work quickly and on ice to minimize RNA degradation during processing. RNA was reverse transcribed into complementary DNA using a reverse transcriptase kit (Takara Biotech, Japan), ensuring that all reagents and equipment were pre-cooled. Quantitative real-time PCR (qRT-PCR) analysis was performed using ChamQ SYBR qPCR Master Mix (Vazyme Biotech Co., Ltd., China) and CFX Manager software (Bio-Rad Laboratories Inc.). GAPDH was used as an internal control to standardize expression levels in each sample. The primer sequences used in this study are detailed in Table 1. Relative expression levels were determined using the 2^−ΔΔCt^ method.

**Table 1:** Primers used in this study

Gene		5′→3′ Sequence
Runx2	Forward	CAGTTCCCAAGCATTTCATC
	Reverse	TCAATATGGTCGCCAAACAG
Type-1 collagen	Forward	TAACCCCCTCCCCAGCCACAAA
	Reverse	TTCCTCTTGGCCGTGCGTCA
GAPDH	Forward	TTGATGTCATCATACTTGGCAGGT
	Reverse	CAG TCAAGGCTGAGAATGGGA
Runx2a masns-isoform	Forward	CTCCCGCTTTAGGACTTCGA
	Reverse	GGAGTCACCGAGCTGAAAAGACT
Collagen 1α2	Forward	GGAAACCTGAAGAAGGCTGTGT
	Reverse	TGAAAGTGAAGCGGCTGTTG
OC	Forward	TGGCCTCTATCATCATGAGACAGA
	Reverse	CTCTCGAGCTGAAATGGAGTCA
Osteopontin (OPN)	Forward	CGCTCAGCAAGCAGTTCAGA
	Reverse	AGAATAGGAGGTGGCCGTTGA
β-actin	Forward	CAACAGGGAAAAGATGACACAGAT
	Reverse	CAGCCTGGATGGCAACGT
MMP3	Forward	CTCCCCACCTTGAATGAAGA
	Reverse	ACTGGGTCGCTTCTCTTGAA
OPN	Forward	AACCCAGACACAAGCATTCC
	Reverse	GCCTTTGAGGTTTTTGGTCA
Mitogen-activated protein kinase (MAPK)	Forward	ATTGTCAGCAATGCATCCTG
	Reverse	ATGGACTGTGGTCATGAGCC
GAPDH	Forward	AGCAATGCTTGTCAATCCTG
	Reverse	ATTGGTCGGACTGATGAGCC

### 2.11 Statistical analysis

For statistical analysis, all quantitative data were presented as mean values with standard deviations. Initially, the Student’s *t*-test was used to compare significant differences between the two experimental groups. For comparisons involving more than two groups, data were subjected to one-way analysis of variance (ANOVA) to evaluate overall group differences. To identify specific differences between groups following the ANOVA, post hoc analysis was performed using Tukey’s multiple comparison test. Statistical significance was set at *P* < 0.05.

## 3 Results

### 3.1 Biocompatibility nature of phytol-micelles: *in vitro* and *in vivo* studies

**Figure 1:**
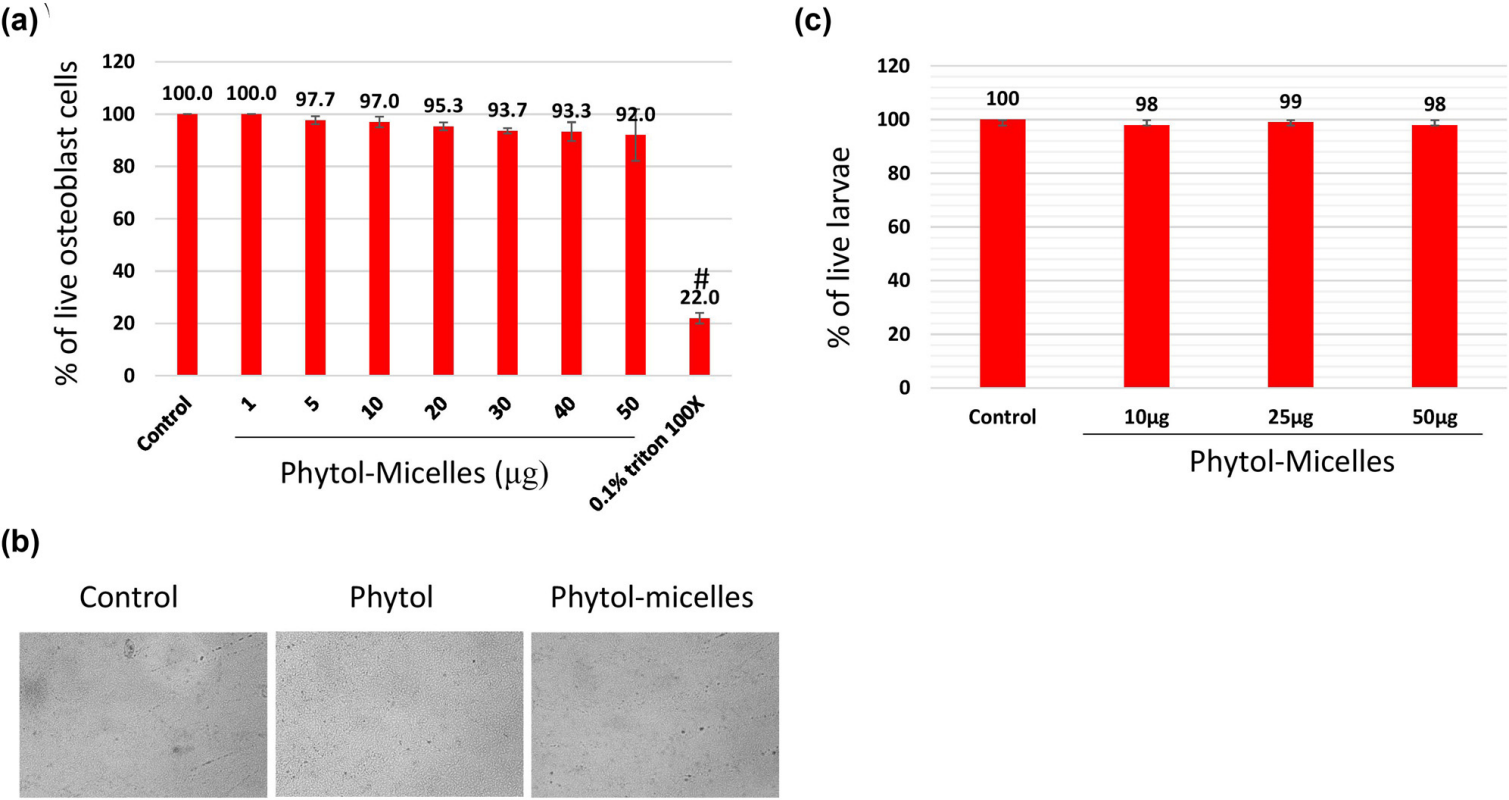
Biocompatibility assessment of phytol-micelles. MG63 cells treated with varying phytol-micelles concentrations (0–50 μg) for 48 h showed no toxicity (a) representative cell image following exposure to 50 μg of phytol-micelles for 48 h. The image shows the cellular morphology and there are no any notable changes induced by the treatment (b). Zebrafish larvae treated with phytol-micelles (50 μg) showed no toxicity (c). # indicates a significant decrease.

To investigate the biocompatibility of phytol-micelles, MG63 cells underwent MTT assay after exposure to various concentrations of phytol-micelles (0–50 μg) for up to 48 h. The results, illustrated in Figure 1a and b, demonstrated a concentration-dependent increase in cellular metabolic activity. Particularly, at a concentration of 25 μg, phytol-micelles significantly enhanced cellular activity compared to the control group. Importantly, no cytotoxic effects were observed even at the highest tested concentration of 50 μM. Furthermore, the *in vivo* biocompatibility of phytol-micelles was assessed using a zebrafish larvae model. Following treatment, the live percentage of larvae was determined, revealing no significant toxicity in zebrafish larvae even at the highest concentration of 50 μg phytol-micelles (Figure 1c). These findings collectively indicate the favorable biocompatibility profile of phytol-micelles both *in vitro* and *in vivo*, supporting its potential utility in osteoblast differentiation and bone formation studies.

### 3.2 Phytol-micelles promote osteoblast differentiation at the cellular level

**Figure 2:**
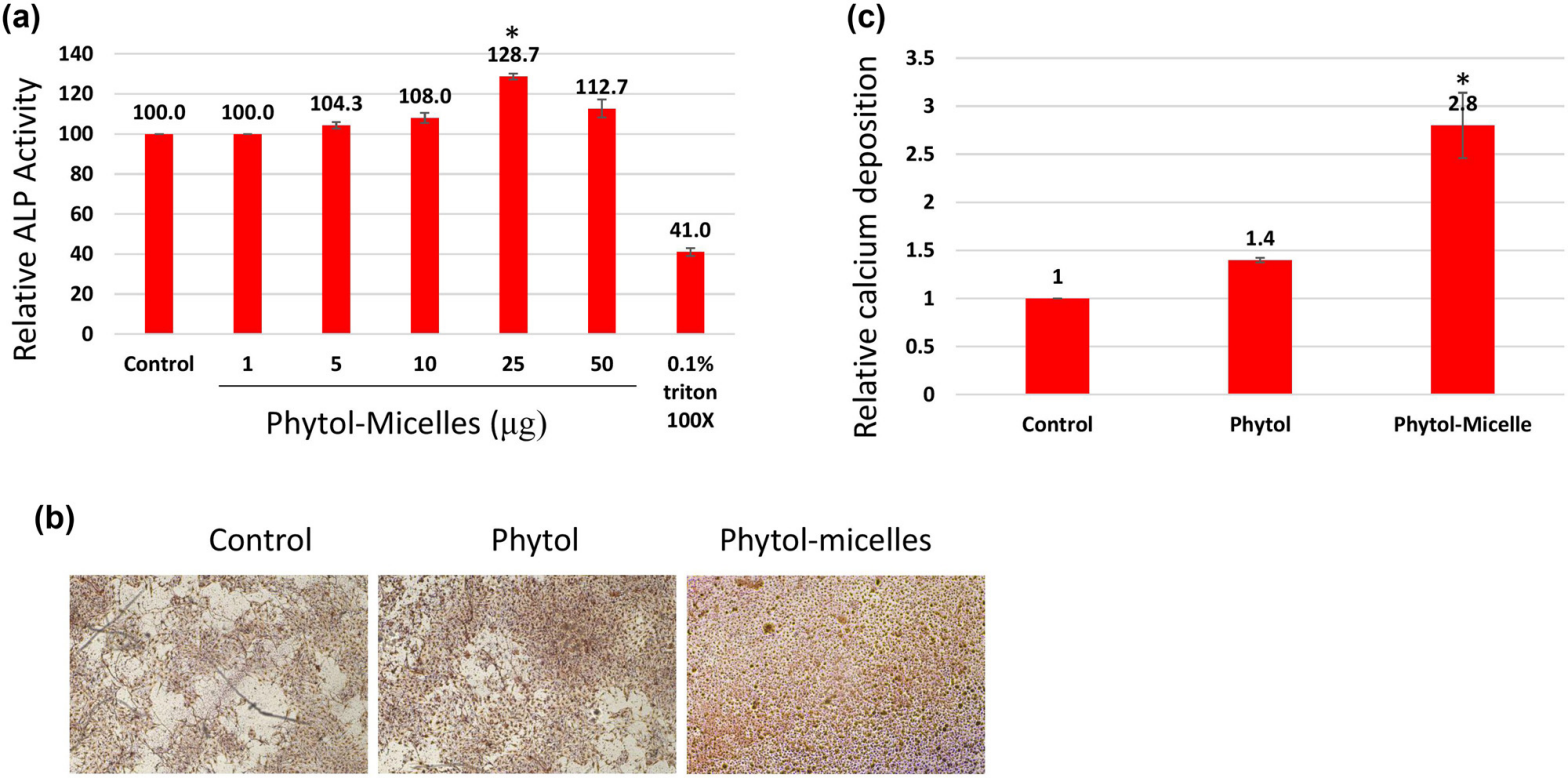
Effects of phytol-micelles on osteoblast differentiation and mineralization. MG63 cells treated with varying phytol-micelles concentrations (5–50 μM) for 7 days showed increased ALP activity, notably higher at 25 μg (a). Treatment with 25 μg phytol-micelles for 14 days enhanced calcium deposition, indicating the potential for osteoblast differentiation and mineralization (b) and quantification (c). * indicates a significant increase.

In this study, we investigated the effects of phytol-micelles on osteoblast differentiation using MG63 cells exposed to varying concentrations (5–50 μM) of phytol-micelles in osteogenic medium. After 7 days of treatment, ALP activity was assessed, revealing that 25 μg of phytol-micelles exhibited notably higher ALP activity compared to other concentrations (Figure 2a). To further elucidate the impact of phytol-micelles on osteoblast mineralization, MG63 cells were treated with 25 μg of phytol-micelles for a period of up to 14 days. Following treatment, the cells were subjected to alizarin staining to evaluate calcium deposition qualitatively (Figure 2b) and quantitatively (Figure 2c). These findings suggest a potential role for phytol-micelles in enhancing osteoblast differentiation and mineralization, particularly at the concentration of 25 μg.

### 3.3 Phytol-micelles promote osteoblast differentiation at the molecular level

**Figure 3:**
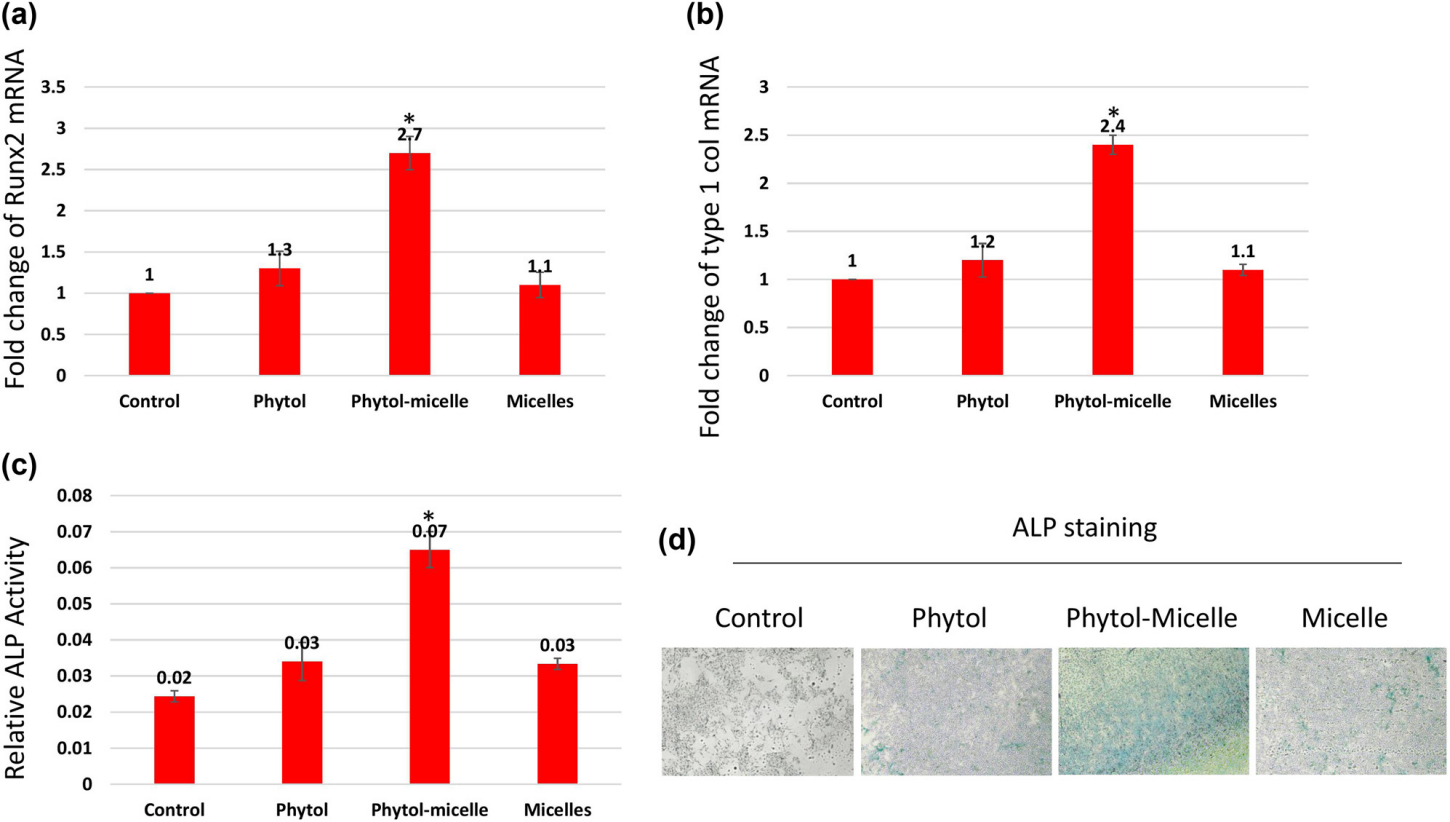
Molecular mechanisms underlying phytol-micelles effects on osteoblast differentiation. MG63 cells treated with 25 μg phytol-micelles in an osteogenic medium for 7 days showed increased mRNA expression of Runx2 (a) and type 1 collagen (b), along with elevated ALP activity (c) and ALP staining (d). * indicates a significant increase.

**Figure 4:**
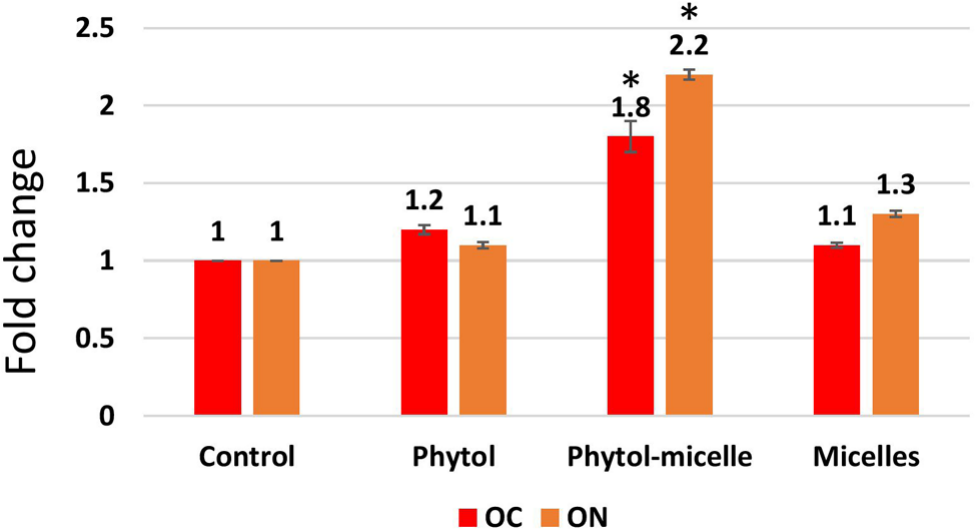
The enhanced secretion of OC and ON was observed. The data illustrate the enhanced levels of these bone matrix proteins, indicating improved bone formation and mineralization. * indicates a significant increase.

To elucidate the molecular mechanisms underlying the effects of phytol-micelles, MG63 cells were treated with 25 μg of phytol-micelles in osteogenic medium for 7 days, and the expression of osteoblast marker genes was analyzed. Initially, the mRNA expression levels of Runx2 (Figure 3a) and type 1 collagen (Figure 3b) were assessed. The results demonstrated a significant increase in the expression of both genes at day 7 compared to the control group, with a further enhancement observed in cells treated with 25 μg of phytol-micelles. Additionally, ALP activity was measured at day 7 (Figure 3c and d), revealing a notable increase in activity following treatment with 25 μg of phytol-micelles. Furthermore, the levels of OC and ON were assessed after 7 days of phytol-micelles treatment (Figure 4). Consistent with the upregulation of osteoblast marker genes and ALP activity, the secretion of OC and ON was significantly enhanced in cells treated with 25 μg of phytol-micelles compared to the control group. These findings collectively indicate that phytol-micelles, particularly at a concentration of 25 μg, exert a stimulatory effect on osteoblast differentiation and molecular markers associated with bone formation.

### 3.4 Effect of phytol-micelles on calcium and phosphorus

**Figure 5:**
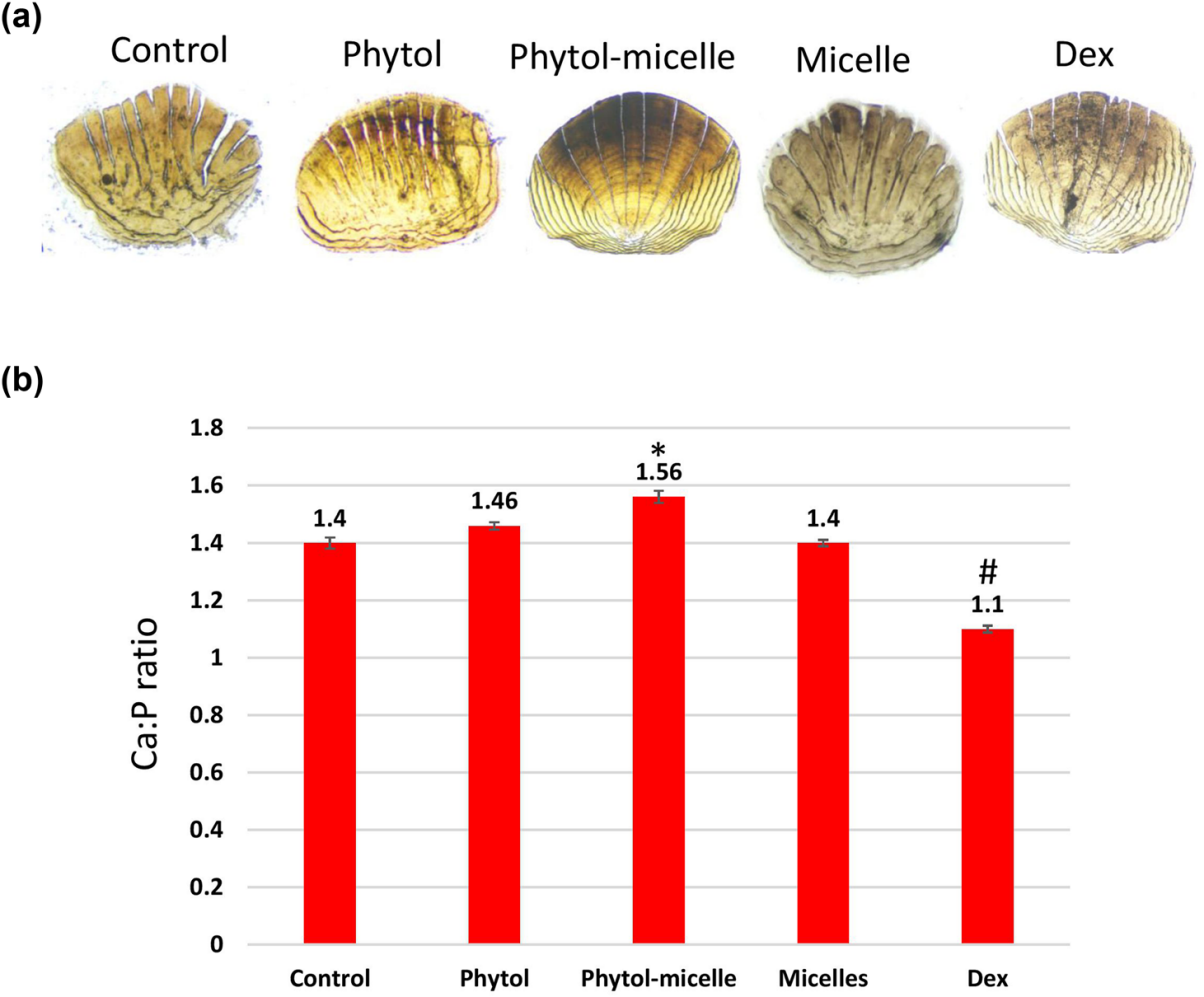
Phytol-micelles influence on calcium and phosphorus levels in zebrafish scales. (a) vonKossa staining of scales and (b) calcium and phosphorus content. * indicates a significant increase. # indicates a significant decrease.

The influence of phytol-micelles on calcium and phosphorus levels was meticulously examined in this study. Initially, the impact of Dex treatment on calcium and phosphorus content was assessed, revealing a significant reduction in both minerals compared to untreated, normal zebrafish, as visually depicted in Figure 5. This decline underscores the deleterious effect of Dex on calcium and phosphorus homeostasis in zebrafish scales. Conversely, upon treatment with phytol-micelles, a remarkable contrast was observed in the calcium and phosphorus levels of regenerated zebrafish scales (Figure 5a and b). Phytol-micelles treatment led to a substantial increase in both calcium and phosphorus content compared to the Dex-treated group, as well as the untreated, normal zebrafish group. This notable enhancement in mineral levels suggests a potential protective and regenerative effect of phytol-micelles on calcium and phosphorus metabolism in zebrafish scales. These findings underscore the promising therapeutic implications of phytol-micelles in mitigating Dex-induced mineral depletion and promoting mineral restoration in zebrafish scales.

### 3.5 Effect of phytol-micelles on HP

**Figure 6:**
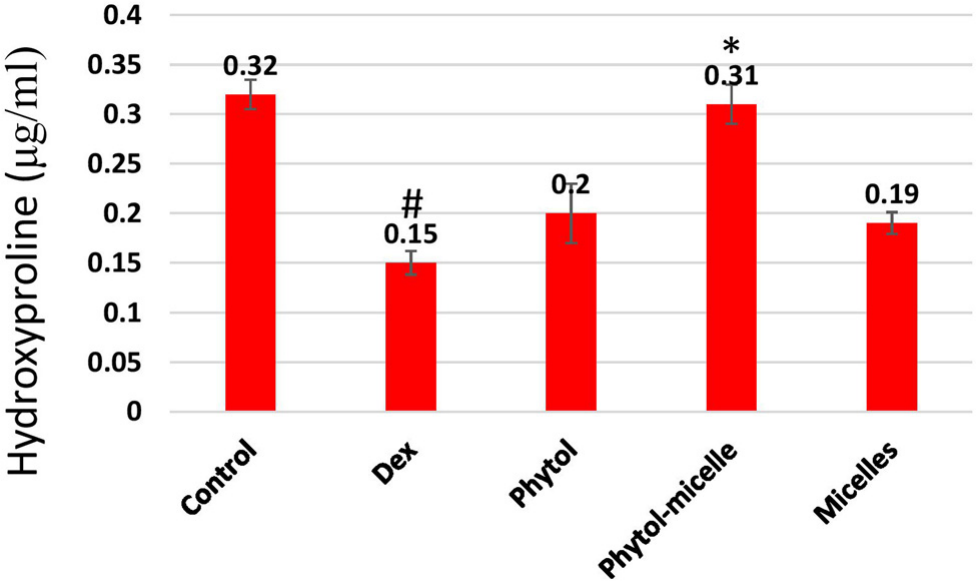
Dex treatment reduces HP levels in zebrafish scales, contrasting with phytol-micelles’ stimulatory effect, as depicted. * indicates a significant increase. # indicates a significant decrease.

The impact of phytol-micelles on HP levels was investigated in this study. As illustrated in Figure 6, treatment with Dex resulted in a significant downregulation of HP levels compared to control zebrafish scales. This decrease highlights the suppressive effect of Dex on HP content in zebrafish scales. Conversely, treatment with phytol-micelles yielded a starkly different outcome, with a significant increase observed in HP levels compared to the Dex-treated group. This elevation underscores the stimulatory effect of phytol-micelles on HP production in zebrafish scales. These findings indicate the contrasting effects of Dex and phytol-micelles on HP levels, with Dex exhibiting a suppressive effect whilst phytol-micelles demonstrates a stimulatory effect (Figure 6). Such observations shed light on the potential therapeutic benefits of phytol-micelles in counteracting Dex-induced suppression of HP and promoting its augmentation in zebrafish scales.

### 3.6 Effect of phytol-micelles on ALP and TRAP activities

**Figure 7:**
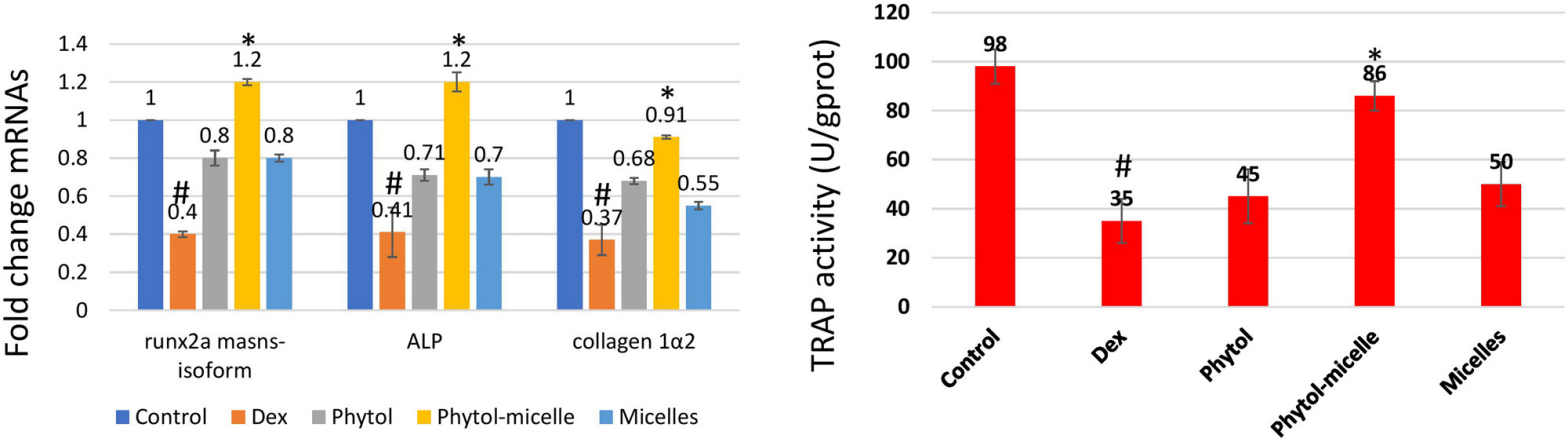
Dex treatment decreases ALP, runx2a mansn-soform, and collagen1a2 mRNAs but increases TRAP activity in zebrafish scales, contrasting with phytol-micelles’ stimulatory effect on ALP and inhibitory effect on TRAP activities, as depicted. * indicates a significant increase. # indicates a significant decrease.

The impact of phytol-micelles on ALP and TRAP activities was assessed in this study. As depicted in Figure 7, treatment with Dex led to a significant decrease in ALP activity compared to the control zebrafish scale. This reduction highlights the inhibitory effect of Dex on ALP activity in zebrafish scales. Conversely, Dex treatment resulted in a notable increase in TRAP activity relative to the control group. This elevation underscores the stimulatory effect of Dex on TRAP activity in zebrafish scales. In contrast, treatment with phytol-micelles yielded opposing outcomes. Phytol-micelles significantly increased ALP activity compared to the Dex-treated group, indicating its stimulatory effect on ALP activity in zebrafish scales. Additionally, phytol-micelles treatment resulted in a significant decrease in TRAP activity relative to the Dex-treated group, highlighting its inhibitory effect on TRAP activity in zebrafish scales. These findings elucidate the differential effects of Dex and phytol-micelles on ALP and TRAP activities, with Dex exerting suppressive effects on ALP activity while promoting TRAP activity, and phytol-micelles exhibiting stimulatory effects on ALP activity while inhibiting TRAP activity in zebrafish scales. Such insights underscore the potential therapeutic benefits of phytol-micelles in modulating bone metabolism parameters and mitigating the adverse effects associated with Dex treatment.

### 3.7 Effect of phytol-micelles on alizarin red staining

**Figure 8:**
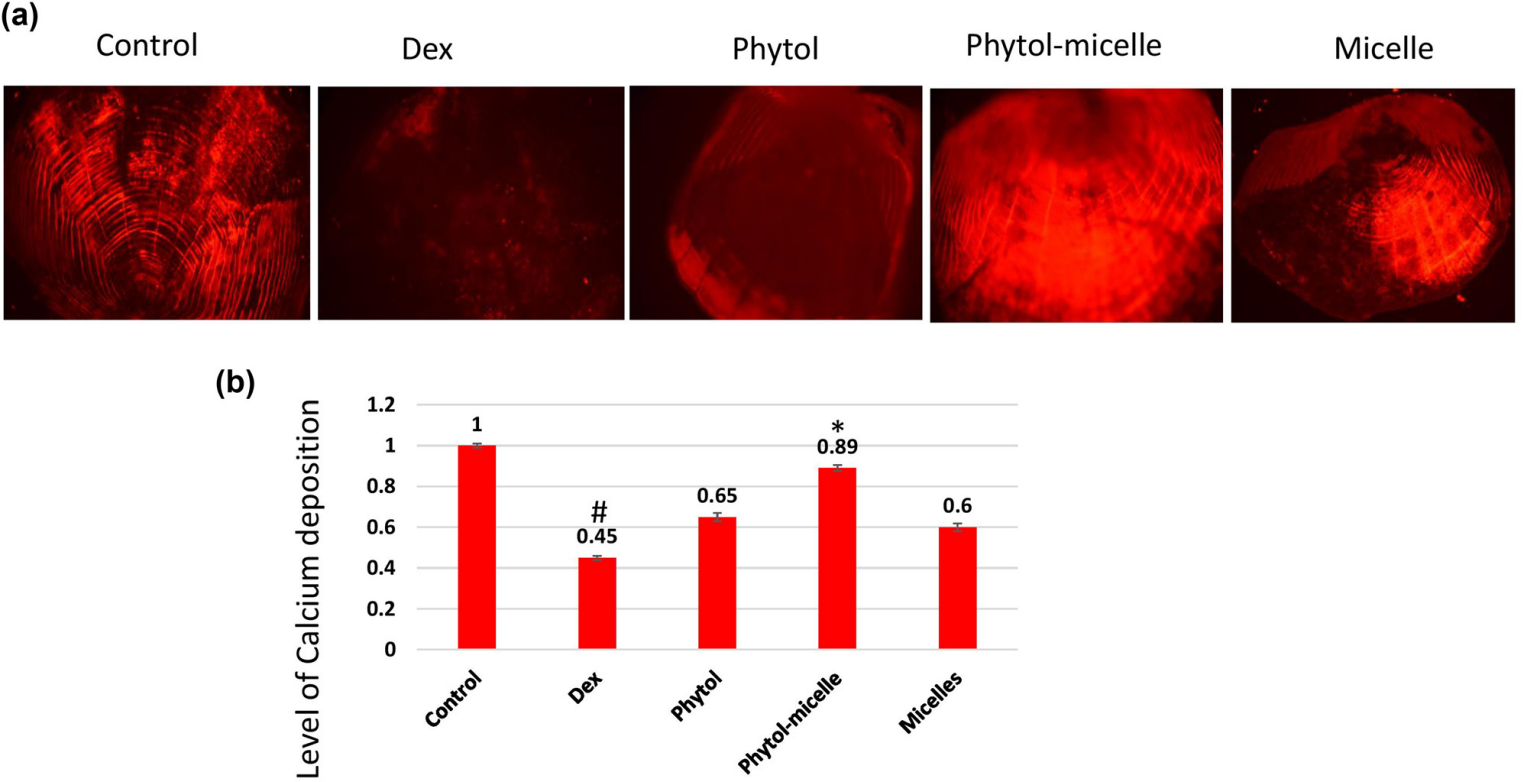
Dex treatment decreases alizarin red staining intensity (a), indicating reduced calcium mineralization (b) in zebrafish scales, contrasting with phytol-micelles’ stimulatory effect on alizarin red staining, suggesting enhanced calcium mineralization. * indicates a significant increase. # indicates a significant decrease.

The impact of phytol-micelles on alizarin red staining, indicative of calcium mineralization, was investigated in this study. As illustrated in Figure 8, treatment with Dex led to a significant reduction in alizarin red staining intensity compared to the normal scales. This decrease suggests the inhibitory effect of Dex on calcium mineralization in zebrafish scales. Conversely, treatment with phytol-micelles resulted in a notable increase in alizarin red staining intensity relative to the Dex-treated group. This elevation highlights the stimulatory effect of phytol-micelles on calcium mineralization in zebrafish scales. These findings underscore the differential effects of Dex and phytol-micelles on calcium mineralization, with Dex exerting suppressive effects while phytol-micelles exhibit stimulatory effects in zebrafish scales. Such insights provide valuable implications for the potential therapeutic use of phytol-micelles in promoting bone mineralization and counteracting the detrimental effects associated with Dex treatment.

### 3.8 Effect of phytol-micelles on MMP3, OPN, and MAPK mRNA levels

**Figure 9:**
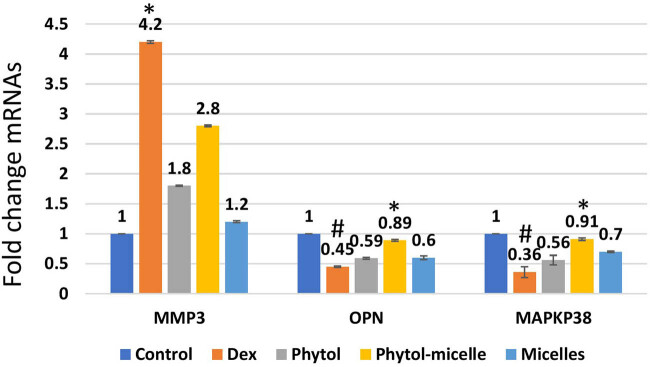
Dex treatment alters mRNA levels, reducing OPN and MAPKp38 while increasing MMP3 in zebrafish scales, indicating suppressive effects. Conversely, phytol-micelles significantly increase OPN and MAPKp38 mRNA levels while reducing MMP3, suggesting stimulatory effects. These molecular changes elucidate phytol-micelles’ potential mechanisms in bone metabolism and remodeling. * indicates a significant increase. # indicates a significant decrease.

The influence of phytol-micelles on the mRNA levels of MMP3, OPN, and MAPKp38 was examined to elucidate its molecular effects in this study. As depicted in Figure 9, treatment with Dex resulted in a significant decrease in the mRNA levels of OPN and MAPKp38, while MMP3 mRNA levels were notably increased compared to the control group. These observations indicate the suppressive effect of Dex on OPN and MAPKp38 expression, accompanied by an upregulation of MMP3 expression in zebrafish scales. In contrast, treatment with phytol-micelles led to a substantial increase in the mRNA levels of OPN and MAPKp38, while MMP3 mRNA levels were significantly reduced relative to the Dex-treated group. These findings underscore the stimulatory impact of phytol-micelles on OPN and MAPKp38 expression, along with its suppressive effect on MMP3 expression in zebrafish scales. Overall, the results suggest that phytol-micelles exerts differential effects on the mRNA levels of MMP3, OPN, and MAPKp38 compared to Dex treatment. Such molecular alterations provide valuable insights into the potential mechanisms underlying the therapeutic effects of phytol-micelles in modulating bone metabolism and remodeling processes.

## 4 Discussion

Osteoporosis is a prevalent systemic bone disorder characterized by diminished bone mass and deterioration of bone tissue microarchitecture, leading to increased bone fragility and fracture susceptibility [16]. With the aging population and escalating incidence of osteoporosis, it poses a significant threat to human health [17]. In this investigation, we utilized Dex to induce an osteoporosis model in zebrafish to assess the pharmacological effects and underlying mechanisms of phytol-micelles against osteoporosis. Bone remodeling, essential for maintaining bone strength and integrity, is a dynamic process influenced by the opposing actions of osteoblasts, responsible for bone formation, and osteoclasts, responsible for bone resorption [18]. Mixed micelles, first proposed by Hoffman and Borgstrom, are formed by a mixture of amphiphilic biliary and dietary components, specifically phospholipids and bile salts. The hydrophobic tails of EPC molecules are positioned in the micellar core to reduce interaction with water molecules, while their polar head groups are located near the water interface. Phospholipids in water typically form bilayered structures; however, the addition of bile salts causes the development of mixed micelles. The micellar structures can dissolve poorly water-soluble medicines in their inner core [19,20,21,22,23,24,25,26,27]. Mixed micelles demonstrate thermodynamic stability, usually retaining sizes ranging from 5 to 60 nm [28]. Small diameters are essential for delivering phytol-micelles to the enterocyte surface and retaining mixed micelles at the base of microvilli. The equilibrium between bone formation and resorption is crucial for bone metabolism, as an imbalance may lead to osteoporosis and increased fracture risk [19]. According to the findings of our research, phytol-micelles demonstrated a significant increase in mineralization inside osteoblastic cells. This was observed at the cellular level. The development and mineralization of osteoblasts are processes that are highly regulated and choreographed by several genes. These genes include ALP, collagen type 1, OC, and ON, and they are controlled by the bone-specific transcription factor Runx2. The treatment with phytol was shown to be related to a considerable increase in the mRNA expression of Runx2 and Type 1 collagen in MG63 cells, according to the findings of our experiment. Furthermore, the levels of OC and ON secretion were significantly increased as a result of the phytol-micelles therapy, with the ON levels exhibiting a particularly marked rise in comparison to the levels associated with OC and the control groups. These data together imply that phytol can enhance osteoblast development and mineralization *in vitro*, possibly through its impact on critical regulatory genes and proteins involved in bone formation. Phytol-micelles has been shown to have this ability.

To evaluate bone metabolism, we measured TRAP activity as a marker of osteoclast activity and ALP activity as a measure of osteoblast differentiation. Our results indicated that Dex significantly decreased ALP and increased TRAP activity compared to the control group, signifying an imbalance favoring bone resorption. Conversely, phytol-micelles treatment significantly increased ALP and decreased TRAP activity, suggesting a restoration of bone formation/resorption equilibrium closer to the control level. HP is an essential amino acid involved in bone remodeling, predominantly present in collagen, a significant constituent of the bone matrix. It contributes significantly to the stabilization of the collagen triple helix structure, which is crucial for maintaining bone strength and integrity. Collagen is produced by osteoblasts and broken down by osteoclasts during bone remodeling, resulting in the release of HP into the circulation. Elevated concentrations of HP in the blood or urine can suggest heightened bone resorption. Therefore, the measurement of HP levels functions as a biomarker for bone turnover and aids in evaluating the efficacy of therapies for bone-related diseases, such as osteoporosis. Its function in preserving the integrity of the bone matrix highlights its significance in bone health [29,30].

Matrix metalloproteinases (MMPs) have emerged as crucial regulators of osteoclast-mediated bone resorption [20]. Among the MMP family, MMP3 plays a pivotal role in degrading type I collagen, facilitating osteoclast activation [21]. Our findings revealed that Dex treatment significantly upregulated MMP3 expression, whereas phytol-micelles treatment markedly downregulated MMP3 expression in zebrafish scales. These results suggest that phytol-micelles may mitigate osteoclast-mediated bone resorption, potentially through MMP3 regulation. OPN, secreted by osteoblasts, plays a vital role in bone mineralization and matrix adhesion [22]. Reduced OPN expression is associated with impaired bone mineralization and osteoporosis [23]. In our study, Dex treatment led to a significant decrease in OPN expression, while phytol-micelles treatment substantially increased OPN expression in zebrafish scales. These findings suggest that phytol-micelles may enhance bone mineralization and osteoblast function by upregulating OPN expression.

The MAPK signaling pathway is intricately involved in regulating osteoblast proliferation, differentiation, and apoptosis [24]. Activation of the MMP3-OPN-MAPK pathway has been implicated in enhancing osteoblast activity and proliferation [25]. Our results demonstrated that Dex treatment significantly decreased MAPK expression, whereas phytol-micelles treatment markedly increased MAPK expression in zebrafish scales. These findings indicate that phytol-micelles may promote osteoblast activity and bone formation by activating the MMP3–OPN–MAPK signaling pathway. This study suggests that phytol-micelles may mitigate osteoporosis by restoring the balance of bone formation and resorption and activating the MMP3–OPN–MAPK pathway. In osteoporosis, dysregulation of the MMP3–OPN–MAPK pathway can lead to increased bone resorption and decreased bone formation. Further investigations are warranted to elucidate the therapeutic potential of phytol-micelles for osteoporosis treatment [31].

These findings highlight the therapeutic potential of phytol-micelles in mitigating Dex-induced osteoporosis in zebrafish. Through a series of *in vitro* and *in vivo* experiments, phytol-micelles demonstrated a significant impact on bone mineralization, osteoblast differentiation, and molecular pathways involved in bone remodeling. Phytol-micelles treatment resulted in elevated calcium and phosphorus levels, increased bone mineralization, and enhanced osteoblast differentiation, as evidenced by ALP activity and alizarin red staining. Moreover, phytol-micelles exhibited a favorable biocompatibility profile both *in vitro* and *in vivo*, further supporting its potential utility in osteoporosis management. At the molecular level, phytol-micelles treatment was associated with the restoration of the MMP3–OPN–MAPK pathway, which plays a crucial role in regulating osteoblast activity and bone formation. By modulating key genes and proteins involved in bone remodeling, phytol-micelles may help restore the balance between bone formation and resorption, thereby mitigating osteoporosis progression. Overall, these findings provide valuable insights into the pharmacological effects and underlying mechanisms of phytol-micelles in osteoporosis treatment. Further research is warranted to explore the translational potential of phytol-micelles-based therapies for human osteoporosis and to elucidate its long-term safety and efficacy profiles. With continued investigation, phytol-micelles holds promise as a potential therapeutic agent for addressing the growing burden of osteoporosis and improving bone health worldwide.

Although zebrafish are a useful model for investigating osteoporosis, there are several significant drawbacks. Their metabolism and bone structure are quite different from those of mammals, which might have an impact on how discoveries are directly applied. Zebrafish, for example, have a distinct scale-based system instead of a genuine bone structure, which could not accurately mimic the mechanisms involved in human bone remodeling. Zebrafish also have distinct metabolic and regulatory processes, which may affect the outcome. By bridging the gap between preclinical findings and clinical relevance, this comparison directs future directions in research. Furthermore, it facilitates the recognition of possible obstacles and modifications required during the transfer of therapeutic approaches from zebrafish to mammals, ultimately leading to more precise and efficacious interventions for osteoporosis in humans [32,33]. To translate these results into clinical applications, future research should focus on conducting clinical trials to evaluate the efficacy and safety of phytol in human subjects. Additionally, long-term safety studies are necessary to assess any potential adverse effects and confirm the sustained benefits of phytol treatment. This comprehensive approach will help establish phytol’s viability as a therapeutic option for osteoporosis.
